# A pilot study on ultrashort peptide with fluconazole: A promising novel anticandidal combination

**DOI:** 10.14202/vetworld.2023.1284-1288

**Published:** 2023-06-09

**Authors:** Rula M. Darwish, Ali H. Salama

**Affiliations:** 1 Department of Pharmaceutics and Pharmaceutical Technology, School of Pharmacy, the University of Jordan, Amman 11942, Jordan; 2 Department of Pharmacy, Faculty of Pharmacy, Middle East University, Amman 11831, Jordan

**Keywords:** *Candida albicans*, drug combinations, fluconazole, synergism, ultrashort peptide

## Abstract

**Background and Aim:**

Human infections caused by *Candida albicans* are common and range in severity from relatively treatable skin and mucosal conditions to systemic, fatal invasive candidiasis. The treatment of fungal infections is challenged by major obstacles, including the scarcity of effective therapeutic options, the toxicity of available medications, and the escalating antifungal resistance. Hence, there exists an urgent need to develop new classes of antimicrobial agents. This study was conducted to investigate the effect of KW-23 peptide against standard and resistant strains of *C. albicans* alone and in combination with fluconazole.

**Materials and Methods:**

A conjugated ultrashort antimicrobial peptide (KW-23) was designed and synthesized. KW-23 was challenged against standard and multidrug-resistant *C. albicans* alone and in combination with fluconazole using standard antimicrobial and checkerboard assays. The toxicity of the peptide was examined using hemolytic assays.

**Results:**

KW-23 positively affected the standard and resistant Candidal strains (at 5 and 15 μg/mL respectively), exhibiting potent synergistic antimicrobial activity against the standard strain when combined with fluconazole. The effect of the combination was additive against the resistant strain (0.6 μg/mL). Furthermore, the peptide exhibited negligible toxicity on human erythrocytes.

**Conclusion:**

KW-23 and its combination with fluconazole could be a promising candidate for developing anticandidal agents.

## Introduction

*Candida albicans* is a yeast that causes fungal infections in animals and humans. Infections most commonly occur in the oral, rectal, and genital regions of the human body. Fungal infections are also common in people with diabetes and other immune disorders. Most people have some form of *Candida* in their body at some point of their life. Nevertheless, it is important to control *Candida* growth before it causes serious disease, as it can result in a high mortality rate of approximately 20%–40% [1, 2]. Fluconazole, a triazole compound, is one of the most commonly prescribed antifungal drugs for *Candida* infections that inhibit ergosterol biosynthesis during antimycotic therapy [3]. *Candida albicans* can develop resistance to fluconazole through mutations in the drug target, changes in the sterol biosynthesis pathway, and gain-of-function mutations in transcription factors that lead to the constitutive upregulation of ergosterol biosynthesis genes and multidrug efflux pumps [4].

Antimicrobial peptides (AMPs) are considered promising prophylactic and treatment alternatives for various infectious diseases. Antimicrobial peptides have been demonstrated to be effective against bacteria, fungi, viruses, and protozoa and are less likely to induce resistance due to their multiple cellular targets [5]. Antimicrobial peptides are small, low-molecular-weight cationic peptides that are part of the innate immune response of most organisms [6–8]. In addition to their antibacterial action, natural and synthetic AMPs can exhibit immunomodulatory activity by modulating inflammation, chemotaxis, immune system, and cell differentiation. Some of these peptides are already in clinical trials [9]. Antimicrobial peptides can act synergistically with conventional antibiotics, improving their antimicrobial activity [10, 11]. In this context, the synergistic action of AMPs associated with current antimicrobial therapy can yield important results for the production of effective novel drugs to overcome antimicrobial resistance.

This study aimed to produce a unique ultrashort AMP (USAMP) with low peptide length and hydrophobic conjugation, which may improve the cytotoxic profile of the peptide by decreasing its hemolytic activity [12, 13]. The antifungal activity of the peptide was evaluated against standard and resistant *C. albicans* strains. We also investigated the effects of peptide–fluconazole combination to determine possible activity improvement while reducing the side effects.

## Materials and Methods

### Ethical approval

Ethical approval was not necessary for this study.

### Study period and location

The study was conducted in December 2022 at the experimental base of the University of Jordan.

### *Candida* strains

Standard strain of *C. albicans* (ATCC 10231) that is susceptible to fluconazole and *C. albicans* fluconazole-resistant strain (ATCC MYA-574).

### Peptide design and synthesis

**Figure-1:**
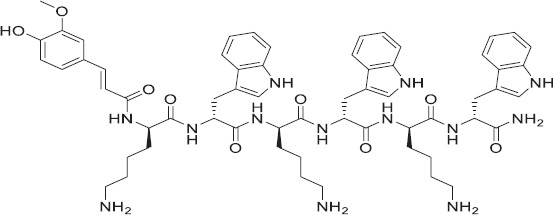
The structure of KW-23 peptide.

The peptide was termed KW-23 and is a hex USAMP consisting of six alternating subunits of lysine and tryptophan conjugated with hydrophobic moieties of ferulic acid. The designed peptide used in this study was obtained from GL Biochem Ltd., Shanghai, China, and was produced using the solid-phase method in a freeze-dried state. Reverse-phase high-performance liquid chromatography was used to purify the peptide using a C18 Internsil^®^ (Thermo Fisher, USA). ODS-SP column, and the column was eluted with an acetonitrile/H_2_O-TFA gradient at a flow rate of 1.0 mL/min. Purification and identification of the synthesized peptide were confirmed by electrospray ionization-mass spectrometry [14]. The design technique relied on providing the peptide a low cationicity using both of the previously described positively charged amino acids [15], which allowed the peptide to connect to the negatively charged membranes of bacterial cells through electrostatic contact. Moreover, to confer the hydrophobicity required for the peptide to permeabilize the target membranes, the peptide was conjugated to ferulic acid. This highly hydrophobic molecule is believed to act as an anchor for peptide–membrane hydrophobic interaction. The net positive charge of KW-23 is +3, and its molecular weight is 1136.34 Da. The structure of the KW-23 peptide is shown in (Figure-1).

### Determination of the minimum inhibitory concentration (MIC) and minimum fungicidal concentration (MFC) of KW-23

All antifungal assays with some modifications were conducted according to the Clinical and Laboratory Standards Institute M27-A3 broth microdilution susceptibility test criteria. Briefly, *C. albicans* cell suspensions at 2 × 10^3^ cells/mL were treated with 100–0.78 μM KW-23 in Sabouraud dextrose agar. The lowest concentration that inhibited clear fungal growth at the end of the incubation period was defined as the MIC. All experiments were conducted in triplicate at separate time points. The MFC was determined by taking 10 μL aliquot from the first clear well after the turbid one, and were streaked on sterile labeled nutrient media agar and then incubated for 24 h at 37°C. Minimum fungicidal concentration (MFC) was determined as the concentration that caused the eradication of 99.9% of viable cells. All experiments were performed in triplicate [16].

### Determination of MIC and MFC of fluconazole

The MIC and MFC of fluconazole were determined against reference *C. albicans* strain (ATCC 10231) and resistant *C. albicans* strain (ATCC MYA-574). For this purpose, different concentrations of fluconazole were prepared by dissolving fluconazole in water and then diluting it in sterile broth.

Minimum inhibitory concentrations and MFC determinations were performed in triplicate [17].

### Determination of fractional inhibitory concentration (FIC) of KW-23 and fluconazole combination

The synergistic activity and MIC values of KW-23 and fluconazole combination were investigated using a previously established micro broth checkerboard assay [18]. The procedure was conducted as described in the previous section with the addition of fluconazole in combination with KW-23 to estimate the MIC of the combination. All experiments were conducted in triplicate. The synergistic action was evaluated using the FIC.

The FIC is the summation of the inhibitory concentration values of each antifungal component in the antifungal combination divided by the inhibitory concentration of the individual antifungal, as shown in the equation below [19].

FICI = (MIC peptide in combination/MIC peptide alone) + (MIC fluconazole in combination/MIC fluconazole)

Synergistic (FIC ≤0.5), additive (FIC 0.5 < FIC ≤1), indifferent (1 < FIC ≤4), or antagonist (FIC >4).

### Hemolytic activity of KW-23

The ability of KW-23 to damage the membrane of normal erythrocytes was evaluated using previously reported erythrocyte hemolytic tests [20].

Every experiment was conducted in triplicate.

The equation for hemolysis:


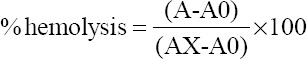


Where A: Is OD 450 with the peptide solution,

A0: Is OD 450 of the blank.

## Results

### *In vitro* antimicrobial activity of KW-23 and fluconazole

The antifungal activities of KW-23 and fluconazole were evaluated against the standard and resistant strains of *C. albicans* (ATCC 10231 and ATCC MYA-574, respectively). Table-1 shows the MIC and MFC values of KW-23 and fluconazole. The MIC values of KW-23 were 5 and 15 μg/mL against the standard and resistance strains, respectively. The MFC values were equal to the MIC values, indicating a fungicidal behavior of KW-23. For fluconazole, the MIC values were 14 and ≥64 μg/mL against the standard and resistance strains, respectively.

**Table-1:** The MIC and MFC values of KW-23 and fluconazole against *Candida albicans*.

Anti-fungal agent	*Candida albicans* (ATCC 10231) MIC*/MFC** (µg/mL)	*Candida albicans (*ATCC MYA-574) MIC*/MFC** (µg/mL)
KW-23	5/5	15/15
Fluconazole	14/65	MIC ≥64 µg/mL

* MIC=Minimum Inhibitory concentration,

** MFC=Minimum fungicidal concentration

### Synergistic activity of KW-23

The synergistic effects of KW-23 with fluconazole were evaluated using the checkerboard method. The values of synergy for KW-23, along with fluconazole, were determined using the FIC index and are shown in Table-2.

**Table-2:** MIC and FIC index of KW-23 and fluconazole against standard and resistant strains of *Candida albicans.*

*Candida* strain	Fluconazole MIC (µg/mL)	Fluconazole synergistic MIC (µg/mL)	KW-23 MIC (µ g/mL)	KW-23 synergistic MIC (µg/mL)	FIC* index	Action
*Candida albicans* (ATCC 10231)	14	5	5	0.025	0.37	Synergistic
*Candida albicans* (ATCC MYA-574)	35	15	15	2.5	0.6	Additive

* FIC=Fraction Inhibitory concentration, MIC=Minimum inhibitory concentrations

### Hemolytic activity of KW-23

KW-23 exhibited no hemolytic action against human erythrocytes up to a concentration of 100 g/mL. Table-3 shows the results of the hemolytic assay.

**Table-3:** The effect of different concentrations of KW-23 on human erythrocytes.

Concentration of KW-23 (µ M)	Hemolysis (%)
0	0
5	0
10	0
20	0
40	0
60	0
80	1
100	3

## Discussion

The emergence of invasive fungal infections has motivated the development of new antifungal medicines due to the significant resistance of *C. albicans* and other *Candida* species to commonly used antifungal drugs, particularly azoles [21, 22]. Antimicrobial peptides may be promising candidates for developing novel antifungal agents due to their broad-spectrum activity against pathogens and their low probability of developing antibiotic resistance [23–25]. It has been demonstrated that AMPs are less susceptible to the development of resistance due to their rapid effect and pharmacodynamic properties compared with conventional drugs [26]. Several natural antifungal peptides such as polymyxin, melittin, and protegrin have demonstrated potent antimicrobial activity, but their therapeutic application is limited due to their toxic effects on erythrocytes [27]. *In silico* peptide optimization is extremely promising to produce new peptides from scratch or enhance existing ones [27]. Additional research on AMPs and their methods of action is required to produce potential antifungal treatments. Designing synthetic and semisynthetic peptides represent an effective and affordable method to reduce the costs associated with discovering and designing antifungal compounds [28].

In this study, KW-23, a synthetic peptide, was produced and its cationicity, hydrophobicity, and hydrophilicity were considered. These conjugated hydrophobic moieties may act as an anchor for peptide–membrane insertion and permeability [29]. Based on the MIC values obtained in this study, KW-23 was much more potent than fluconazole against *C. albicans*, which is consistent with the previous studies demonstrating the potent effects of AMPs [30, 31]. The use of two or more drugs with distinct modes of action in a drug combination therapy can enhance the effectiveness of current antimicrobial therapies. Combination regimens can limit the development of antifungal resistance as demonstrated in monotherapy, increase efficacy, reduce toxic side effects, and reduce the cost of each individual drug [32]. Numerous studies have revealed that AMPs and traditional antifungal agents synergize together [33, 34]. In this study, the KW-23 and fluconazole combination demonstrated synergistic activity when tested against the *C. albicans* strain ATCC 10231. Moreover, the combination exhibited an additive effect against the *C. albicans* strain ATCC MYA-574. In general, two substances that interact synergistically most probably exhibit antimicrobial activity through a different mechanism of action. Fluconazole is an azole-based antifungal agent that is fungistatic and interferes with the primary enzyme involved in ergosterol biosynthesis, preventing the production of ergosterol. Regarding AMPs, several different mechanisms of action have been proposed, including the capacity of AMPs to harm cell membranes, interact with intracellular targets such as by inhibiting cell wall, nucleic acid, and protein synthesis, and induce apoptosis [35]. The fact that the peptide synergistically interacted with fluconazole in our study suggests that its mode of action is distinct from that of fluconazole, and the synergistic effects may be explained by the fusion of the two distinct mechanisms of action. The peptide KW-23 designed in this study exhibited low toxicity to erythrocytes with a low hemolytic effect, probably due to the overall low hydrophobicity.

## Conclusion

The designed peptide KW-23 demonstrated promising activity against *C. albicans*. It also exerted a synergistic effect when used in combination with fluconazole, a conventional antifungal agent, and therefore, KW-23 may reduce the probability of resistance development in *C. albicans* against the conventional drug. Further, research is necessary to elucidate the mechanism of action of the peptide.

## Authors’ Contributions

RMD: Conceptualization and supervision. RMD and AHS: Investigation, methodology, and writing – review and editing. All authors have read, reviewed, and approved the final manuscript.
